# NF-κB inactivation in myeloid cell leads to reprogramming of whole-body energy metabolism in response to high-fat diet

**DOI:** 10.1038/s41420-025-02659-7

**Published:** 2025-08-05

**Authors:** Xianghong Wang, Zhe Yang, Xin Ye, Owen P. McGuinness, Hengwen Yang, Hongyun Lu, Jianping Ye

**Affiliations:** 1 Department of Endocrinology and Metabolism, Zhuhai People’s Hospital (Zhuhai Clinical Medical College of Jinan University), Zhuhai, China; 2 Pennington Biomedical Research Center, Louisiana State University, Baton Rouge, Louisiana USA; 3 Department of Molecular Physiology and Biophysics, Vanderbilt University Medical Center, Nashville, Tennessee USA; 4 Guangdong Provincial Key Laboratory of Tumor Interventional Diagnosis and Treatment, Zhuhai Institute of Translational Medicine, Zhuhai People’s Hospital (Zhuhai Clinical Medical College of Jinan University), Jinan University, Zhuhai, China; 5 Institute of Trauma and Metabolism, Zhengzhou Central Hospital Affiliated to Zhengzhou University, Zhengzhou, China; 6 Tianjian Laboratory of Advanced Biomedical Sciences, Academy of Medical Sciences, Zhengzhou, China

**Keywords:** Mechanisms of disease, Metabolic diseases

## Abstract

Energy metabolism is subject to reprogramming in the body upon bacterial or virus infection. It is generally believed that immune cells sense the stress signals of infection to mediate the reprogramming of whole-body energy metabolism. However, the key molecules required for the immune cell function remain to be identified. In this study, we addressed the issue by examining the energy metabolism in Lyz2-p65-KO mice, in which p65 (RelA) gene is inactivated in myeloid cells. On Chow diet, the p65-KO mice exhibited no difference to the wild type mice in the energy metabolism. On a high fat diet (HFD), the KO mice gained less adipose tissue and body weight for improved insulin sensitivity and blood lipids along reduction in pro-inflammatory cytokine. This was observed with more energy loss in feces. The KO mice showed a reduction in metabolic rate after LPS challenge for accelerated decrease of oxygen consumption. They had a high mortality rate in the septic shock model with less elevation of serum pro-inflammatory cytokines and more elevation of anti-inflammatory cytokines. In vitro, the KO macrophages expressed less pro-inflammatory cytokines in response to stimulation by palmitic acid, IL-1β and TNF-α. In conclusion, the data suggest that p65 is a key molecule in myeloid cells to mediate the reprogramming of energy metabolism under stress conditions of HFD feeding.

## Introduction

Myeloid cells including macrophages and granulocytes are activated upon bacterial or virus infection to orchestra the immune reactions [1]. In non-infectious conditions, they are activated by chronic inflammation in adipose tissue related to obesity [2, 3]. In both scenarios, the NF-κB pathway is activated intracellularly to facilitate their functions in the inflammatory response. NF-κB acts as a transcription factor, influencing gene expression through activation or repression. It has been reported that major vault protein, as a suppressor for NF-κB signaling in macrophages, suppresses obesity and atherosclerosis through inhibiting IKK-NF-κB signaling mediated inflammation [4]. However, subsequent studies have indicated that IKKβ-KO mice exhibit increased inflammasome activity and enhanced inflammatory responses under endotoxin stimulation [5]. NLRP3 inflammasome senses danger signals associated with obesity and participates in obesity-induced inflammation and insulin resistance [6]. NF-κB-induced p62 expression promotes mitochondrial autophagy or may inhibit the synthesis of new DNA in mitochondria [7]. Studies have demonstrated that NF-κB plays a crucial role in controlling inflammation in adipose tissue, our previous study indicated that NF-kB activity was found to protect the brown adipocytes through down-regulation of adenine nucleotide translocase 2 (ANT2) in the control of apoptosis [8]. These findings suggest that NF-κB may limit and promote inflammatory responses through the expression and secretion of cytokines in myeloid cells. However, the impact of NF-κB activity on systemic energy metabolism remains unknown, as these published studies have not thoroughly addressed this issue.

Endotoxin is considered one of the main triggers of chronic inflammation in the pathogenesis of obesity, similar to the effects of hypoxia in adipose tissue [9, 10]. It has been reported that the concentration of endotoxin in the blood of obese mice is increased due to enhanced absorption from the colon [11]. However, in most studies, elevated levels of pro-inflammatory cytokines (such as TNF-α and IL-1β) have not been detected in the blood of obese humans or rodents, despite increased mRNA expression of these cytokines in some tissues associated with obesity. The role of endotoxin remains to be examined [12]. Endotoxin is a recognized stimulus that induces pro-inflammatory cytokines in circulation. The effects of lipopolysaccharide (LPS) are readily detectable in patients and rodent models under conditions of septic shock. Given that LPS-induced inflammatory responses can be exacerbated by obesity, such effects should be detectable in obese conditions [6].The reasons behind this contradiction are still unclear. When assessing the endotoxin activity associated with obesity-related inflammation, a sensitive model is required.

In this study, we addressed the above issues in Lyz2-p65-KO mice (KO mice). These KO mice exhibited defects in energy response to HFD and LPS for a reduction in energy storage and mobilization. A significant reduction in IL-1β was detected after LPS stimulation. In vitro experiments showed that p65-KO macrophages expressed less pro-inflammatory cytokines.

## Results

### Lyz2-p65-KO mice exhibit less weight and fat tissue in the HFD

**Fig. 1:**
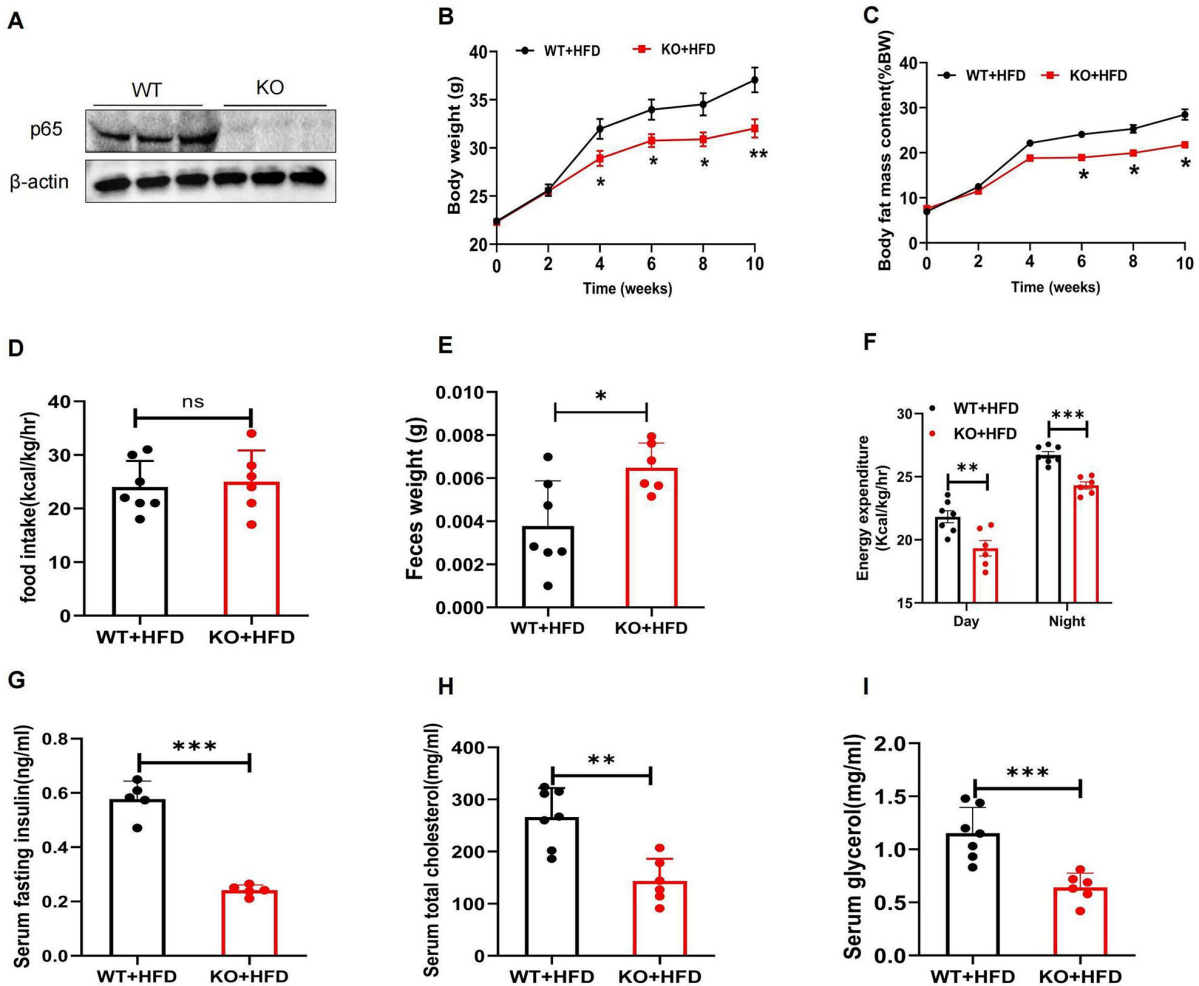
Lyz2-p65-KO mice was protected from obesity by reduction in intestine energy absorption. **A** p65 protein in primary cultured peritoneal macrophage. Proteins were determined by western blotting. **B–I** Comparison of metabolic indicators between WT and Lyz2-p65-KO mice in a high-fat diet. Body weight (**B**), body fat mass content (**C**), food intake (**D**), feces weight (**E**), energy expenditure (**F**), serum fasting insulin (**G**), serum cholesterol (**H**), serum triglycerides (**I**). Data are expressed as mean ± SEM (*n* = 6–7 per group). *P < 0.05, **P < 0.01, ***P < 0.001. ns: no significance.

The Lyz2-p65-KO mice were generated by crossing p65-floxed mice with mice expressing the Cre recombinase under the control of the Lys-Cre promoter. Wild-type (WT) and KO mice were identified by genotyping (Fig. S1A), and the reduced levels of P65 protein and mRNA were confirmed in KO mice macrophages by Western blot and qPCR (Fig. 1A). The KO mice and WT mice were fed on chow diet (CD) and the high-fat diet (HFD), respectively. Under the chow diet, there were no differences in body weight (Fig. S1B), body composition (Fig. S1C), food intake (Fig. S1D), insulin tolerance (Fig. S1E), glucose tolerance (Fig. S1F), oxygen consumption (Fig. S1G), carbon dioxide emissions (Fig. S1H), physical activity (Fig. S1I), energy expenditure (Fig. S1J), and inflammatory factors (Fig. S1K, L) between the KO and WT mice. These data indicate that the inactivation of p65 in macrophages does not alter the metabolic parameters and systemic inflammatory status of mice on the Chow diet. After 12 weeks of HFD feeding on mice, compared with WT mice, the KO mice showed significantly reduction in body weight compared to WT mice (Fig. 1B), a significantly lower fat mass content to body weight ratio (Fig. 1C), and less fat tissue accumulation that was not the result of altered food intake (Fig. 1D), but rather a consequence of energy loss in feces (Fig. 1E). This suggests that the energy absorbed by the gut system is lower. The lower consumption of KO mice measured in the metabolic cage during both the day and night supports this possibility (Fig. 1F). Additionally, KO mice exhibited lower serum insulin levels (Fig. 1G) and significant reductions in serum cholesterol and triglycerides (Fig. 1H, I). These data suggest that Lys-p65-KO mice are partially protected from diet-induced obesity due to reduced energy absorption in the gut.

### Lyz2-p65-KO mice exhibited less insulin resistance in the HFD

**Fig. 2:**
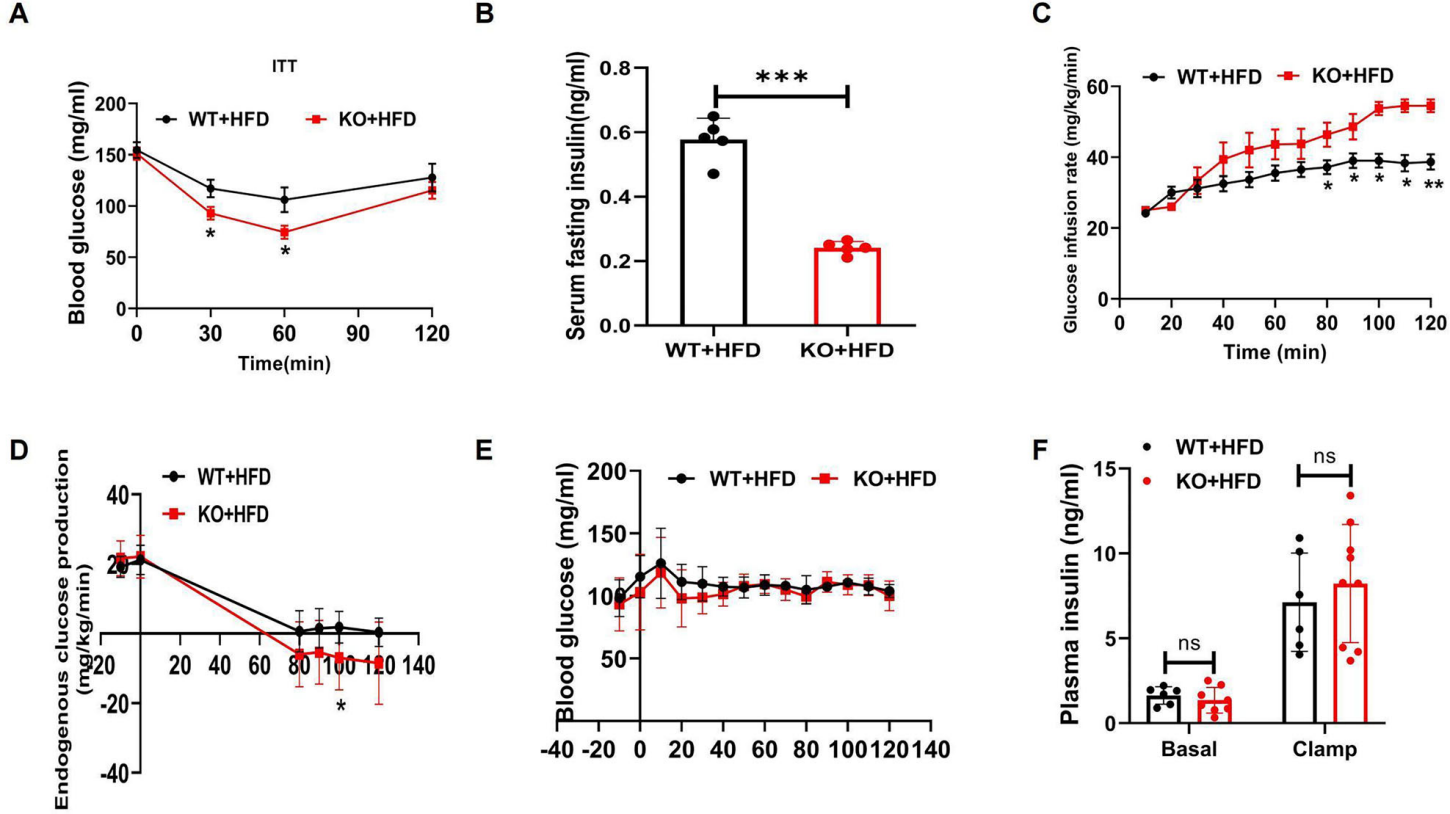
Insulin sensitivity was protected in obese Lyz2-p65-KO mice. Hyperinsulinemic-euglycemic clamp was conducted in conscious mice following 12 weeks of high-fat diet. **A** ITT results of mice on high-fat diet. **B** Plasma fasting insulin level. **C** Steady-state glucose infusion rates (GIR) during clamp experiment. **D** Endogenous glucose production before and during the clamp test. **E** Blood glucose level during clamp experiment. **F** Plasma insulin concentration at base and after clamp test. Data are the means ± SEM (*n* = 6–7 per group). *P < 0.05, **P < 0.001, ***P < 0.001. ns no significance.

On HFD, the KO mice were assessed for insulin sensitivity using the insulin tolerance test (ITT) and euglycemic-hyperinsulinemic clamp technique. The KO mice exhibited better insulin sensitivity with improved insulin tolerance (Fig. 2A), reduced fasting insulin levels (Fig. 2B), and increased glucose infusion rates (Fig. 2C). In the clamp, the KO mice showed a significant reduction in hepatic glucose output (Fig. 2D). Compared to the WT mice, the KO mice had no differences in blood glucose and plasma insulin levels before and after the clamp procedure (Fig. 2E, F). The results suggest that Lyz2-p65-KO mice exhibit better systemic insulin sensitivity on HFD.

### Lyz2-p65-KO macrophages exhibited an anti-inflammatory phenotype

**Fig. 3:**
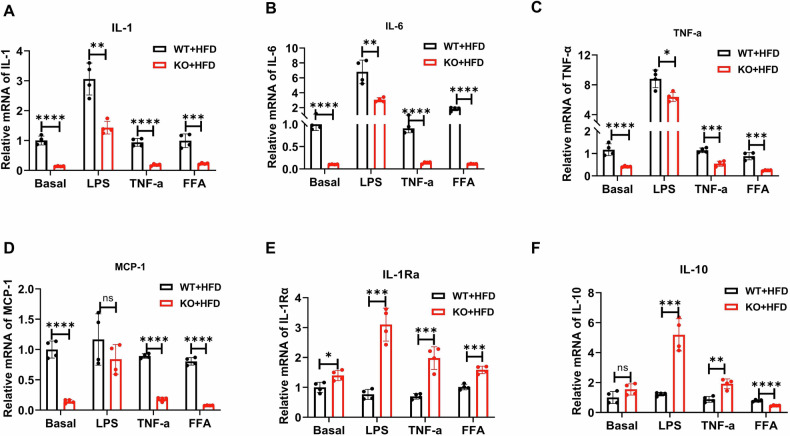
Expression of pro-inflammatory cytokines were decreased and anti-inflammation cytokines were increased in Lyz2-p65-KO mice macrophage. **A**–**F** Primary macrophages were isolated and treated with LPS (10 ng/mL), TNF-α (10 ng/mL) and FFA (300 μmol/L) for 2 h respectively, then RNA was extracted for RT-PCR to detect the mRNA expressions of IL-1 (**A**), IL-6 (**B**), TNF-α (**C**), MCP-1 (**D**), IL-1Ra (**E**) and IL-10 (**F**). Data are the means ± SEM. *P < 0.05, **P < 0.001, ***P < 0.001. ns no significance.

Primary peritoneal macrophages were isolated from WT and KO mice fed with HFD, and cultured in vitro to evaluate the inflammatory responses. In the KO mice, mRNA expression of pro-inflammatory genes including IL-1β, IL-6, monocyte chemoattractant protein-1 (MCP-1), and TNF-α were significantly reduced, while expression of anti-inflammatory genes of IL-1Ra and IL-10 were upregulated (Fig. 3A–D). The gene expression of inflammatory factors was analyzed under stimulation with lipopolysaccharide (LPS, 100 ng/ml), TNF-α (10 ng/ml), and free fatty acids (FFA, 300 μmol/L). At 2 h of stimulation, expression of the four pro-inflammatory cytokines (IL-1β, TNF-α, IL-6, and MCP-1) was decreased, while the expression of two anti-inflammatory cytokines (IL-10 and IL-1Ra) was elevated in the KO mice (Fig. 3E, F). These results demonstrate that p65-deficient macrophages exhibit attenuated pro-inflammatory responses and enhanced anti-inflammatory responses.

### Lyz2-p65-KO mice suffered low metabolic rate in LPS-induced sepsis

**Fig. 4:**
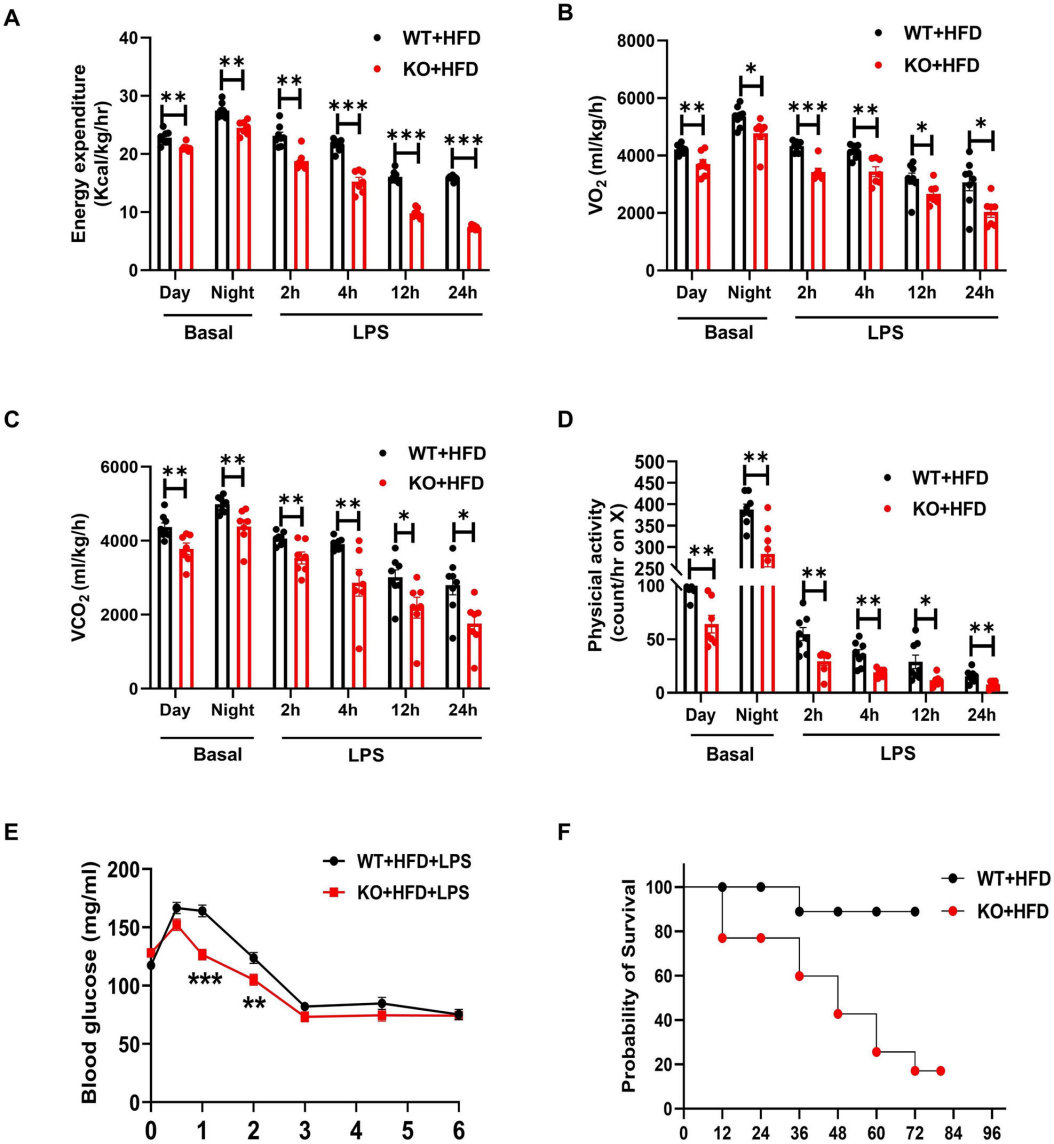
In a high-fat diet, Lyz2-p65-KO mice was more susceptible with LPS challenge than WT mice. **A–E** Mice were placed in metabolic cages and their basal metabolic parameters were recorded. On the 4th day, all mice were intraperitoneally injected with 1 mg/kg LPS, and then the data in the metabolic cages were analyzed at 2, 4, 12 and 24 h. Energy expenditure (**A**). Oxygen consumption (**B**). Carbon dioxide emission (**C**). Physical activity (**D**). Blood glucose after LPS challenge. (**E**). Survival curve (**F**). Data are the means ± SEM (*n* = 5–6 per group). *P < 0.05, **P < 0.001, ***P < 0.001. ns no significance.

Energy metabolism was examined in the WT and KO mice in the basal condition and septic shock condition induced by a non-lethal dose of LPS. In the basal condition, the KO mice exhibited a significant reduction in metabolic rate for a decrease in oxygen consumption rate and carbon dioxide production rate without a reduction in physical activity (Fig. 4A, D). Following intraperitoneal LPS administration to induce septic shock. The KO mice exhibited more reduction in energy expenditure for a larger decrease in oxygen consumption rate and carbon dioxide production rate without a reduction in physical activity (Fig. 4A, D). Subsequently, a glucose tolerance test (GTT) was performed. The results showed that after LPS injection, the KO mice exhibited significantly better glucose tolerance following glucose loading compared to the WT mice (Fig. 4E). These findings indicate that the improvement in insulin resistance and reduced energy expenditure due to the lack of p65 may be at least partially attributed to the attenuation of inflammatory cytokine production. In the 72 h post injection, 80% of the KO mice died, whereas the mortality rate in the WT control group was 20% (Fig. 4F). These data suggest that the energy expenditure was reduced in the KO mice for the increased mortality rate.

### p65-KD macrophages expressed less pro-inflammatory cytokines in response to challenge

**Fig. 5:**
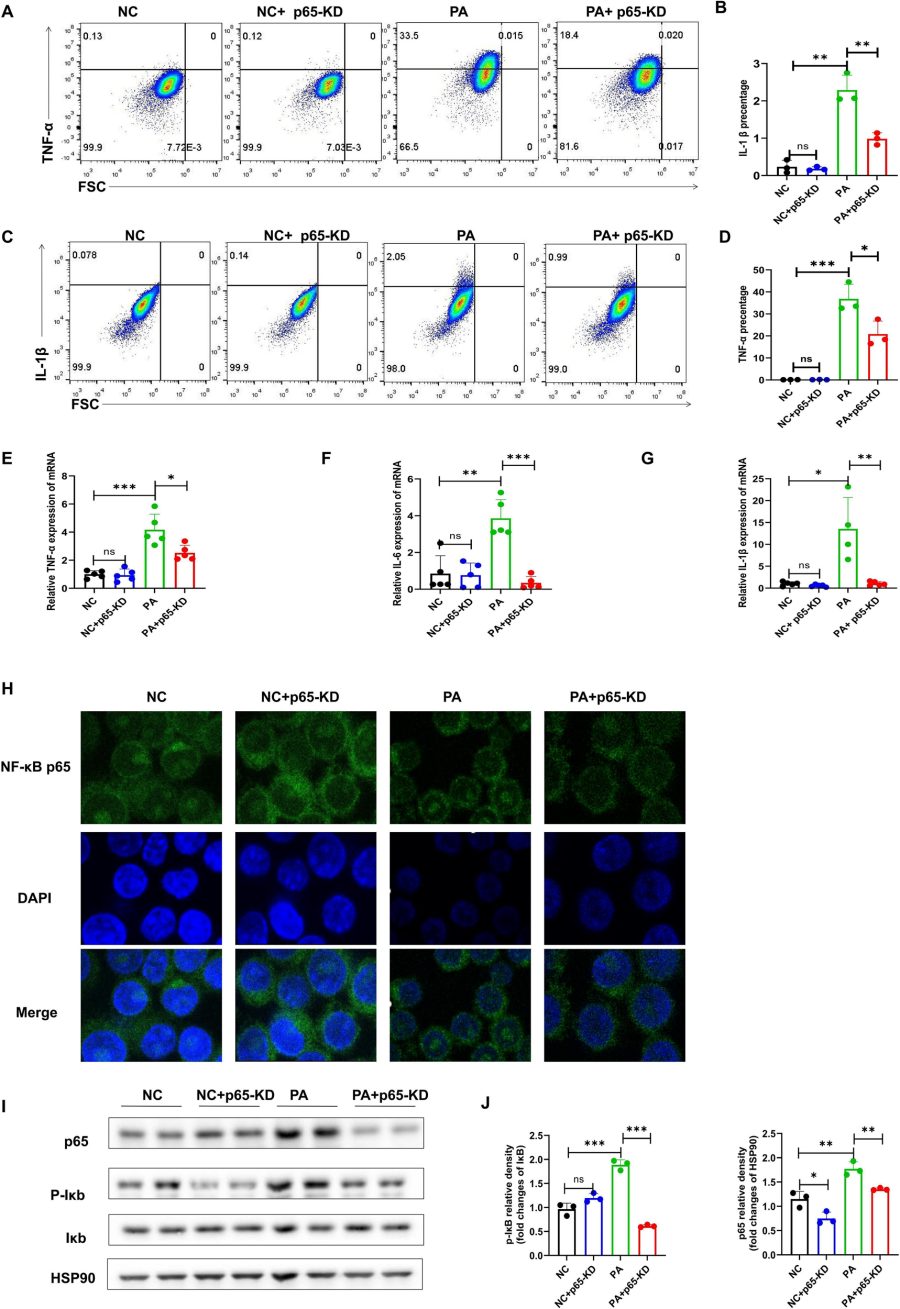
NF-κB p65 is involved in palmitate-induced inflammation in macrophages. **A**–**D** Macrophage inflammatory cytokines were analyzed by flow cytometry when p65 was deficient and PA was exposed. **E**–**G** qPCR were used to detect the levels of inflammatory factors in PA-treated cell media and macrophages, respectively. **H** Changes of NF-κB P65 nuclear translocation. **I**, **J** The effects of raw264.7 induced by PA and p65 inhibited on the activation of NF-κB p65 were analyzed by western bolt and statistical maps. Data are expressed as mean ± SEM. *P < 0.05, **P < 0.001, ***P < 0.001. ns no significance.

In order to investigate the role of p65 in macrophages further, p65 gene knockdown by siRNA was used to reduce p65 activity in cultured RAW264.7 macrophages. Inflammatory response was examined in the p65 knockdown (p65-KD) macrophages after palmitate (PA 0.5 mM) challenge. While PA treatment induced protein expression of inflammatory cytokines (TNF-α, IL-1β, and IL-6), this induction was significantly attenuated in p65-KD cells (Fig. 5A, D). Corresponding reductions were also observed at the mRNA level of these cytokines (Fig. 5E, G). Fluorescence staining data and western blot data showed that P65-KD significantly reduced the p65 protein in the nuclei of macrophages induced by PA (Fig. 5H, J).

### NF-κB p65 regulates the inflammatory response of macrophages induced by pa through NLRP3

**Fig. 6:**
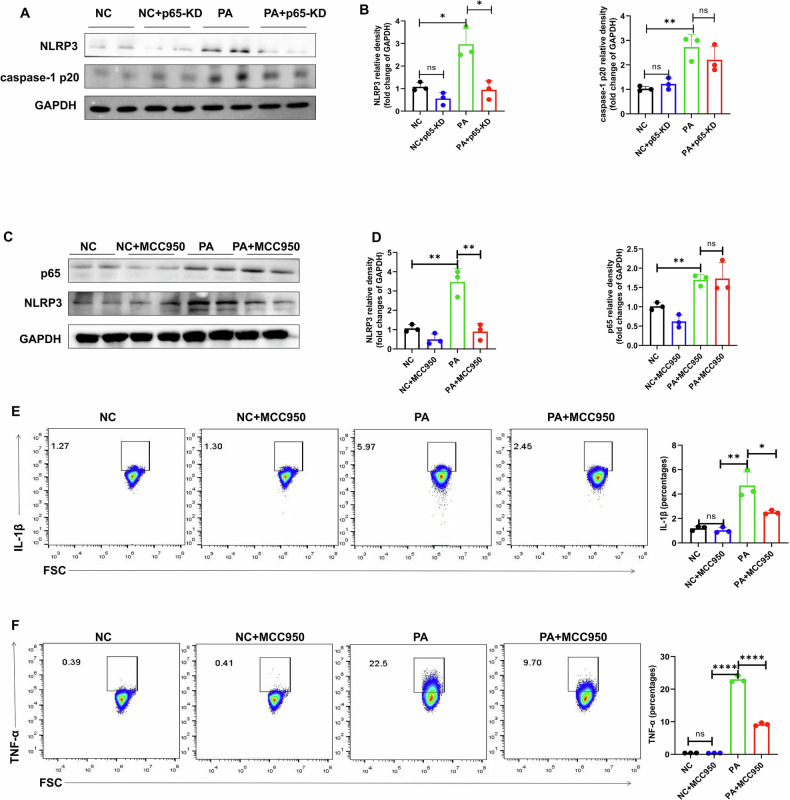
NF-κB p65 regulates PA-triggered macrophage inflammation in relation to NLRP3 activity. **A**–**D** Western blot was used to detect the protein levels of NLRP3, caspase-1 in Raw264.7 cells after p65 knockdown. **E**, **F** Flow cytometry was used to detect the levels of inflammatory cytokines were treated by MCC950 in the presence of PA. Data are expressed as mean ± SEM. *P < 0.05, **P < 0.001, ***P < 0.001. ns: no significance.

In PA-induced macrophages, NLRP3 and caspase-1 protein expressions were found to be elevated, and P65 knockdown significantly decreased the expressions of NLRP3 and caspase-1 proteins (Fig. 6A, B). To further determine whether NLRP3 is a key molecule mediating the effect of NF-κB p65 on inflammatory cytokines under PA attack, NLRP3 inhibitor (MCC950) was found to partially reverse the PA-mediated increase in inflammatory cytokines (Fig. 6C, D).

## Discussion

Obesity has adverse effects on nearly all physiological functions of the human body and poses a significant threat to public health. The mechanisms underlying its development are not yet fully understood. In this study, we focused on the NF-κB p65 transcription factor family, a well-known checkpoint, to explore the role of obesity. We revealed the mechanisms by which macrophage NF-κB activity contributes to diet-induced obesity-related energy metabolism. Through a transgenic mouse model, the phenotype of Lyz2-p65-KO mice indicated that the bone marrow NF-κB pathway is essential for energy expenditure in mice under both physiological and pathological conditions. Energy expenditure involves the activation of the body’s defense mechanisms in response to stress in physiological states. Under basal conditions, Lyz2-p65-KO mice exhibited reduced energy expenditure on a high-fat diet (HFD). This reduction extended into states of septic shock. KO mice demonstrated extreme sensitivity to LPS, with significantly increased mortality under low-dose LPS stimulation (1 mg/kg), and energy expenditure in these mice decreased even further after administration of IL-1β antibody. In cell experiments, it was confirmed that NF-κB inhibits LPS-induced IL-1β-mediated inflammatory responses through NLRP3.

We demonstrated that the absence of the NF-κB p65 subunit in macrophages can prevent dietary-induced obesity and improve insulin resistance. Furthermore, under high-fat diet feeding and LPS stimulation, diet-induced metabolic inflammation was significantly reduced, and the GTT levels in NF-κB KO mice were increased after LPS stimulation. Additionally, palmitic acid (PA) induced a metabolic stress state in macrophages, leading to increased NF-κB expression, nuclear localization, and transcriptional activity, while also elevating NLRP3 inflammation. Knockdown of NF-κB p65 improved PA-induced inflammation by reducing NLRP3 levels. These results provide strong experimental evidence for the critical role of macrophage NF-κB in obesity.

Previous studies found that high-fat diet increased the number of CD11b^+^ F4/80^+^ macrophages in muscle compared with thin mice [13]. During the process of obesity, macrophages modulate the inflammatory status of muscle tissue by secreting cytokines and chemokines [14]. Chronic inflammation disrupts the balance between fat synthesis and breakdown in adipose tissue, leading to elevated circulating free fatty acids and ultimately culminating in muscle inflammation [15]. The secretion of inflammatory factors by macrophages can induce fat breakdown, resulting in heightened lipid metabolism, which is closely associated with skeletal muscle insulin resistance [16]. In this study, we demonstrated increased muscle insulin sensitivity in KO mice using a hyperinsulinemic-euglycemic clamp test. Therefore, the mechanism by which macrophage NK-κB p65 loss improves muscle insulin sensitivity may be related to inhibiting the production of inflammatory factors.

IKKβ is a key molecule in activating NF-κB in the inflammatory response [17]. Previous studies have shown that overexpression of IKKβ in adipose tissue can protect mice from diet-induced weight gain [18], while specific loss of IKKβ in liver cells preserves liver insulin responsiveness but produces insulin resistance in muscle and fat. Myeloid IKKβ KO mice maintained overall insulin sensitivity and were protected from insulin resistance [4]. These results suggest that the IKKβ/NF-κB pathway has different roles in insulin target tissues. Our study shows that macrophage NF-κB inactivation enhances systemic insulin sensitivity in diet-induced obese mice, an observation consistent with the phenotype of mice with previously reported loss of myeloid IKKβ activity [4].

In this study, GTT was improved in NF-κB KO mice after the administration of LPS, suggesting that NF-κB loss in macrophages improves insulin resistance by reducing inflammation. Inflammatory factors produced by macrophages (TNF-α, IL-6, and IL-1β) have been shown to affect lipolysis and cause insulin resistance [19–21]. TNF-α affects lipid metabolism by inducing lipolysis, inhibiting free fatty acid uptake and lipoprotein lipase activity [22]. Studies have shown that the administration of TNF-α can increase the metabolism of free fatty acids by 60% in patients [23], while the levels of free fatty acids are reduced in obese mice lacking TNF-α, demonstrating that TNF-α affects lipid metabolism by inducing lipolysis and inhibiting the uptake of free fatty acids [24]. IL-6 promotes lipolysis and fatty acid oxidation in both mice and humans [25]. One study found that the infusion of IL-6 caused the muscle to release fatty acids, causing an increase in fatty acid levels throughout the body, possibly due to the role of directly regulating skeletal muscle fatty acid metabolism [26]. In addition, IL-1β indirectly stimulates fat breakdown and fatty acid oxidation by affecting the protein activity of fat breakdown, increasing the release of free fatty acids and glycerol [27]. Due to the possibility of increased levels of lipid metabolites leading to insulin resistance [28], these studies suggest that inhibiting inflammatory factors can improve insulin resistance.

Our study also found that NF-κB improves PA-induced inflammation through NLRP3. Under the influence of PA, macrophages accumulated in the tissue begin to produce pro-inflammatory cytokines, which lead to insulin resistance [29]. PA damages the mitochondria by decreasing membrane potential and increasing ROS production, resulting in the release of mitochondrial DNA into the cytoplasm [30], which leads to NLRP3 inflammasome activation.PA may ameliorate inflammation by triggering activation of the NLRP3 inflammasome [31], which is consistent with our study.

While our study provides evidence for the role of p65 in macrophage-mediated inflammation and energy metabolism through in vitro experiments and metabolic phenotyping, we acknowledge that direct in vivo validation of inflammatory pathway activation and macrophage infiltration is essential. Future studies will include immunohistochemical staining and flow cytometry analysis of metabolic tissues (e.g., adipose tissue and liver) to further validate these key findings.

In conclusion, our data indicate that NF-κB p65 inactivation in macrophages improves systemic insulin resistance by reducing the inflammatory response. Inhibition of NF-κb p65 in macrophages may be used to treat insulin resistance.

## Material and methods

### Animal models and treatment

As previously mentioned, the p65-floxed mice were generated on a C57BL/6 genetic background [32]. Lyz2-Cre mice in the C57BL/6 genetic background (Stock # 004781) were purchased from Jackson Laboratory (Bar Harbor, ME). Lyz2-p65-KO (p65^f/f^ Cre^+/−^) mice were generated by crossing the floxed-p65 mice with Lyz2-cre mice. Floxed-p65 littermates (p65^f/f^) were used as wild-type control for the knockout mice. Male mice were used in this study at 8-10 weeks of age. All of the mice were housed in the animal facility at the Pennington Biomedical Research Center with a 12 h light-dark cycle and constant temperature (22 ~ 24 °C), free access to water and diet. The mice were fed Chow diet (11% calories in fat, 5001, Labdiet, St. Louis, MO) or the high-fat diet (HFD, 58% calories in fat, D12331, Research Diets, New Brunswick, NJ). HFD feeding started in mice at 12 weeks in age to generate a diet-induced obese model. All procedures were performed in accordance with the National Institutes of Health guidelines for the care and use of animals and were approved by the Institutional Animal Care and Use Committee (IACUC) at the Pennington Biomedical Research Center.

### General condition, body composition and energy expenditure

As previously mentioned, body composition (fat mass, lean mass, total body water, and free water) was measured using quantitative nuclear magnetic resonance (Minispec Mn10 nuclear magnetic resonance scanner) [33]. Food intake was determined in individually housed mice using a wired cage. The test was conducted daily for 3 days. The intake was determined by the net reduction in diet weight after excluding the spilled diet. Food intake was expressed in g/mouse/day. Feces weight was monitored daily for 3 days and normalized with the body lean mass (kg) and 24 h. Energy metabolism was monitored in mice after 4 weeks on HFD, using the indirect calorimetry system (Comprehensive Laboratory Animal Monitoring System, Columbus Instruments, Columbus, OH). Mice were kept in the metabolic chamber for 6 days. Oxygen consumption (VO_2_), carbon dioxide production (VCO_2_), spontaneous physical activity and food intake were recorded daily. Energy expenditure (EE: kcal/kg/h) was calculated with data on day 5 using the formula EE = [3.815 + 1.232 × VCO_2_/VO_2_] × VO_2_ × 0.001 as described in our published study [8]. Energy expenditure data was normalized with body lean mass.

### Glucose tolerance and insulin sensitivity tests

Following a period of abstinence lasting 4 h, the mice received an intraperitoneal injection of insulin (0.75 units/kg body weight) to undergo the insulin tolerance test (ITT). Subsequently, following an overnight fast, the mice were intraperitoneally administered glucose (2.5 g/kg body weight) to undergo the glucose tolerance test (GTT). Blood glucose levels were assessed at 0, 30, 60 and 120-minute intervals utilizing the FreeStyle blood glucose monitoring system (TheraSense, Phoenix, AZ). Data were expressed in blood glucose concentration (mg/dl).

### Hyperinsulinemic-euglycemic clamp

After the 8-week-old mice were fed either a high-fat diet or chow diet for 12 weeks, a silicone catheter was inserted into the right external jugular vein of the mice to provide an intravenous injection route, with the catheter connected to an intravenous injection access point placed on the back of the mice’s neck. The high-insulin euglycemic clamp experiment was conducted according to the previously outlined protocol [34]. In brief, after fasting for 6 h, the intravenous injection route was established via the access point. During the experiment, capillary blood glucose levels were measured using the tail nick method and a handheld glucometer.

### Biochemical measurement and cytokines assay

Serum insulin was determined using an ELISA kit (catalog number: EZRMI-13K, Millipore). Serum triglycerides (TAG) and glycerol were determined using the Serum Triglyceride Determination Kit (TR0100; Sigma-Aldrich), while total cholesterol was assessed using an assay kit (TR13421, Thermo Fisher Scientific Inc.) following the manufacturer’s instructions. The cytokine assay was performed in a couple of conditions. In response to LPS, blood was collected after 2 hr LPS injection. Intraperitoneal injection of lipopolysaccharide (LPS) at a dose of 1 mg/kg was administered. Plasma was isolated and used in cytokine assays for interleukin-6 (IL-6), interleukin-10 (IL-10), interleukin-1β (IL-1β), monocyte chemoreceptor protein-1(MCP-1) and tumor necrosis factor (TNF-a) using a multiplex kit (MCYTOMAG-70K, Millipore).

### Primary peritoneal macrophage and cell lines

The primary peritoneal macrophages were isolated from mice according to the procedure described elsewhere [35]. Briefly, 2% sterile starch (85643, Sigma) solution was injected into the peritoneal cavity once to induce yield of macrophages. The peritoneal macrophages were harvested 3 days later with 20 mL of cold PBS by lavage and then cultured in RPMI 1640 (supplemented with 10% FBS and 50 µg/ml gentamicin). Cells were treated with LPS (10 ng/ml), TNF-α (10 ng/ml), and free fatty acid (linoleic salt, 300 μmol/L) for 2 h, followed by collection for RT-PCR and protein blotting.

RAW 264.7 maintained in culture medium containing 10% fetal bovine serum and 1% penicillin-streptomycin at 37 °C with 5% CO_2_ humidity. The cells were cultured under two different conditions: bovine serum albumin (B2064; Sigma-Aldrich) without fatty acids served as the control, and 0.5 mM palmitic acid (PA; P9767; Sigma-Aldrich) was cultured for 24 h.

### Transfection with small interfering RNA

In the siRNA knockdown experiment, RAW264.7 cells were transfected with p65 siRNA or negative control siRNA (provided by Puzon Technology Company, Guangzhou, China). Transfection was performed using Lipofectamine 3000 (G04003, GenePharma, Suzhou, China) according to the manufacturer’s protocol. After 72 h of transfection, the knockdown efficiency was assessed by Western blot analysis.

### Immunofluorescence staining

Coverslip-grown cells were fixed in 4% paraformaldehyde, permeabilized in 0.1% Triton X-100, and blocked with 1% bovine serum albumin. The cells were incubated overnight at 4 °C with primary anti-NF-κB p65 antibody, followed by incubation with FITC-conjugated secondary antibody at room temperature for 1 h. Cell nuclei were stained using DAPI (4’,6-diamidino-2-phenylindole). Image acquisition and analysis using confocal laser microscopy (Leica).

### Flow cytometry

For flow cytometric assays, staining was performed according to our previously published protocol [36, 37]. In short, for intracellular cytokine staining, First, the cells were incubated at 37 °C for 4–6 h in RPMI containing 10% FBS, PMA (50 ng/mL), ionomycin (1 µg/mL), and Golgi-Plug (1 µg/mL). After incubation, surface staining was performed, fixed for 30 min, intracellular antibody staining was performed, FACS was performed using Cytek Northern Light™ (Cytek, San Diego, CA, USA), and data was analyzed using Flowjo 10.0 software.

### Quantitative real-time RT-PCR

TRIzol (Invitrogen) reagent was used to extract total RNA from cell or tissue samples, RNA was then reverse transcribed to cDNA synthesis using the PrimeScriptTM RT reagent Kit (RR047A; Takara Biotechnology, Japan), real-time PCR was performed using TB Green Premix Ex Taq™ (RR820A, Takara Biotechnology, Japan). The results were normalized to GAPDH mRNA expression. The primers used in this study are listed in Table S1.

### Western blot analysis

Total proteins were extracted from tissues and cell lysates in RIPA buffer. After centrifugation at 4 °C for 12,000 r/min for 15 min, the supernatant was taken and determined by BCA Protein Assay Kit (Beyotime). Electrophoresis was conducted on an SDS-PAGE gel, followed by transfer to a PVDF membrane, blocked for 1 h with 5% milk, and incubated with primary antibody overnight at 4 °C. This was followed by incubation with an HRP-conjugated secondary antibody for 1 h. The protein signals were detected using an ECL kit (Thermo Scientific, MA, USA). Densitometry analysis was performed using the ImageJ software. The antibodies used were listed in Table S2.

### Statistical analysis

All results are presented as the means ± SEM. Student’s *t* test was employed to assess the significance of variances between two groups, while ANOVA was utilized for multiple comparisons. Statistical significance was defined as *P* < 0.05. Statistical analyses were conducted using GraphPad Prism 8 (GraphPad, La Jolla, CA, USA).

## Supplementary information

clean,supplementary material.

Figure S1

## Data Availability

The data that support the findings of this study are available on request from the corresponding author.
