# Supplementary material for: NF-κB inactivation in myeloid cell leads to reprogramming of whole-body energy metabolism in response to high-fat diet
Source: Cell Death Discov. 2025 Aug 5;11:367. doi: 10.1038/s41420-025-02659-7 (PMC12325682; doi:10.1038/s41420-025-02659-7)
Supplement: Supplementary file 1 — clean,supplementary material. [file 41420_2025_2659_MOESM1_ESM.docx]

| Primer | Sequence (5’— 3’) |
| --- | --- |
| IL-1β | F: TCGCAGCAGCACATCAACAAG |
|  | R:TCCACGGGAAAGACACAGGTAG |
| TNF-α | F: CTGAACTTCGGGGTGATCGG |
|  | R:GGCTTGTCACTCGAATTTTGAGA |
| IL-6 | F: CTGCAAGAGACTTCCATCCAG |
|  | R：AGTGGTATAGACAGGTCTGTTGG |
| P65 | F: ATGACATCCAGATTCGGTT |
|  | R: AGGTCTGATTTCCTCCGAAG |
| GAPDH | F: TGACCTCAACTACATGGTCTACA |
|  | R: CTTCCCATTCTCGGCCTTG |
| MCP-1 | F: GAGGACAGATGTGGTGGGTTT |
|  | R: AGGAGTCAACTCAGCTTTCTCTT |
| IL-10 | F: GCTGGACAACATACTGCTAACC |
|  | R: ATTTCCGATAAGGCTTGGCAA |
| IL-1Ra | F: GCTCATTGCTGGGTACTTACAA |
|  | R: CCAGACTTGGCACAAGACAGG |

Table S1. Primers for quantitative real-time PCR

| Antibody | Product number | Manufacturer |
| --- | --- | --- |
| IκBα antibody | 4814S | Cell Signaling Technology |
| Phospho-IκBα antibody | 2859S | Cell Signaling Technology |
| NF-κB p65 antibody | 8242S | Cell Signaling Technology |
| Phospho-NF-κB p65 antibody | 3033S | Cell Signaling Technology |
| anti-β-actin antibody | AC026 | Abclonal |
| HSP90 | 4874 | Cell Signaling Technology |
| GAPDH | 5174 | Cell Signaling Technology |
| insulin receptor β | sc-81466 | Santa Cruz |
| p insulin receptor β | Sc81501 | Santa Cruz |
| pAkt Ser473 | kp24001 | Calbichem |
| pIRS-1 Tyr 632 | sc-17196 | Santa Cruz |
| NLRP3 | kp24001 | Calbichem |
| Cleaved-Caspase 1 | AF4005 | Affinity Biosciences |

Table S2. Reagents

**Figure legends**

**Figure S1. Macrophages specific NF-κB p65 knockout doesn't impair the metabolic indexes and inflammatory factor in mice on a chow diet.**

**A:** Identification of genotype of NF-κB p65. **B-L:**Comparison of metabolic indicators between WT and Lyz2-p65-KO mice in a chow diet. Body weight (**B**), body lean mass content (**C**), food intake (**D**), ITT (**E**) , GTT (**F**) , oxygen consumption (VO_2_ and VCO_2_)(**G-H**), physicial activity (**I**), energy expenditure(**J**), and inflammatory factor expression (**K-L**) between WT and KO mice on a chow diet. Data are expressed as the mean ± SEM.**P* < 0.05, ***P* < 0.01, ****P* < 0.001. ns: not significant.
