# Supplementary figures and images for: NF-κB inactivation in myeloid cell leads to reprogramming of whole-body energy metabolism in response to high-fat diet
Source: Cell Death Discov. 2025 Aug 5;11:367. doi: 10.1038/s41420-025-02659-7 (PMC12325682; doi:10.1038/s41420-025-02659-7)

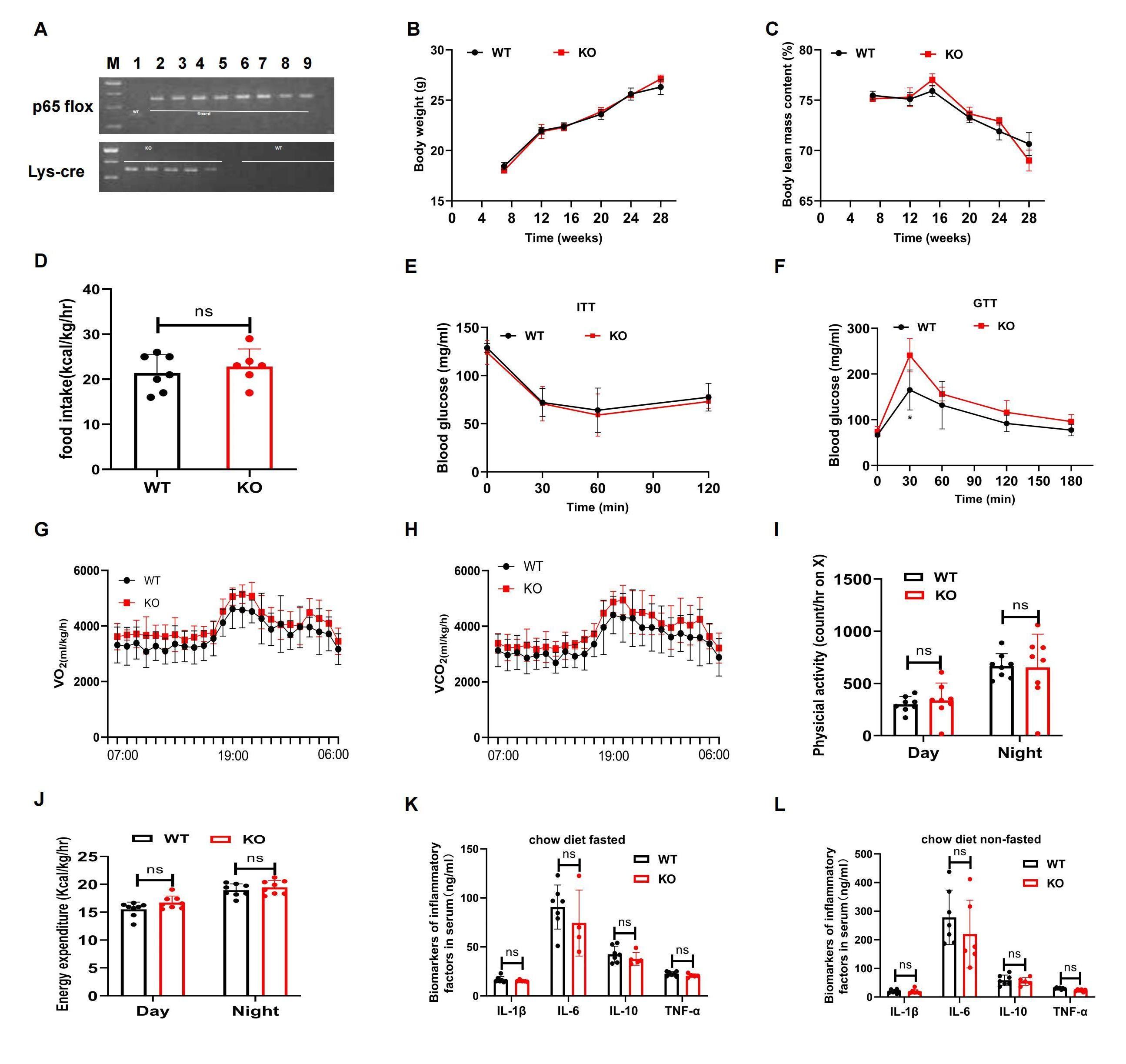

Supplement: Supplementary file 2 — Figure S1 [file 41420_2025_2659_MOESM2_ESM.jpg]
